# From multimodal features to behavioural inferences: A pipeline to model engagement in human-robot interactions

**DOI:** 10.1371/journal.pone.0285749

**Published:** 2023-11-08

**Authors:** Soham Joshi, Arpitha Malavalli, Shrisha Rao

**Affiliations:** Department of Computer Science, International Institute of Information Technology Bangalore, Bangalore, Karnataka, India; Zayed University, UNITED ARAB EMIRATES

## Abstract

Modelling the engaging behaviour of humans using multimodal data collected during human-robot interactions has attracted much research interest. Most methods that have been proposed previously predict engaging behaviour directly from multimodal features, and do not incorporate personality inferences or any theories of interpersonal behaviour in human-human interactions. This work investigates whether personality inferences and attributes from interpersonal theories of behaviour (like attitude and emotion) further augment the modelling of engaging behaviour. We present a novel pipeline to model engaging behaviour that incorporates the Big Five personality traits, the Interpersonal Circumplex (IPC), and the Triandis Theory of Interpersonal Behaviour (TIB). We extract first-person vision and physiological features from the MHHRI dataset and predict the Big Five personality traits using a Support Vector Machine. Subsequently, we empirically validate the advantage of incorporating personality in modelling engaging behaviour and present a novel method that effectively uses the IPC to obtain scores for a human’s attitude and emotion from their Big Five traits. Finally, our results demonstrate that attitude and emotion are correlates of behaviour even in human-robot interactions, as suggested by the TIB for human-human interactions. Furthermore, incorporating the IPC and the Big Five traits helps generate behavioural inferences that supplement the engaging behaviour prediction, thus enriching the pipeline. Engagement modelling has a wide range of applications in domains like online learning platforms, assistive robotics, and intelligent conversational agents. Practitioners can also use this work in cognitive modelling and psychology to find more complex and subtle relations between humans’ behaviour and personality traits, and discover new dynamics of the human psyche. The code will be made available at: https://github.com/soham-joshi/engagement-prediction-mhhri.

## 1 Introduction

Humans are said to display *engaging behaviour* in a human-robot interaction if they are attentive and display an intention to continue the conversation [1]. Predicting human engagement is of interest in various settings—students using educational tools [2], elderly or impaired people interacting with assistive robotics [3], consumers using commercial chat-bots [4, 5], etc. Bohus and Horvitz [6] did pioneering work in using multimodal social signals (gaze, facial expression, language, etc.) to predict engaging behaviour. Following this, the works by Castellano et al. [7] and Foster et al. [8] also focused on the accurate prediction of engaging behaviour directly using multimodal data from sensors. However, direct prediction of behaviour (including engagement) from a few minutes of multimodal data is a primitive approach. Much research [9, 10] shows that utilizing personality inferences in behaviour prediction makes a model more comprehensive, especially in human-robot interaction (HRI). This is due to the relatively static nature of a human’s personality as opposed to behaviour. However, using personality to model behaviour is not fully explored, as indicated by Salam et al. [11]:

“Despite its importance, there have been relatively few works focusing on engagement and/or its relationship to personality in social interaction settings.”

The few existing approaches [12, 13] do not exploit the various theories of social psychology that model behaviour based on various influences. Our work fills the gap. First, we directly predict engagement from multimodal data. Subsequently, we show the immense advantage of incorporating personality and interpersonal behavioural models into predicting engaging behaviour. Finally, we present a pipeline based on Triandis’ Theory of Interpersonal Behaviour to model engagement from multimodal features, incorporating personality inferences. The Triandis Theory of Interpersonal Behaviour (TIB) [14] is a popular behavioural model used to predict various human behaviours, like consumer behaviour [15], entrepreneurial behaviour [16], acceptance [17], etc.

The Multimodal Human-Human-Robot Interaction (MHHRI) dataset [18] comprises six modes of data obtained from sensors during triadic human-human-robot interactions. The required multimodal features have to be extracted from the time-synchronised sensor data. For extraction of visual features, state-of-the-art techniques utilize deep learning architectures like VGG-Face [19], FaceNet-1 [20], etc.

As a baseline experiment on the MHHRI dataset, Celiktutan et al. [18] used the features extracted from the dataset to predict engagement directly. They also predicted personality in the form of the ‘Big Five’ personality traits. The Big Five describe the personality of an individual across five dimensions, namely *extraversion*, *agreeableness*, *openness*, *conscientiousness*, and *neuroticism* [21].

We improve upon the engagement prediction approach of Celiktutan et al. [18] firstly using just the multimodal features and secondly in conjunction with predicted Big Five personality traits. We extract physiological features and first-person vision features from the MHHRI dataset. The accuracy of our predictions for the Big Five traits using multimodal features is better than Celiktutan et al. [18], especially in HRI settings.

In their work, Salam et al. [11] also incorporated the Big Five scores obtained from the MHHRI dataset, along with other multimodal features to improve the predictions of engaging behaviour. To better demonstrate the benefit of including personality in the pipeline, we quantify the correlation between Big Five traits and engaging behaviour. Unlike the work by Salam et al. [11], we don’t stop at incorporating Big Five traits into the pipeline; we also introduce an interpersonal behavioural model. We show that this further improves the accuracy and strengthens the pipeline to predict engaging behaviour. The interpersonal behavioural model that we use is Triandis’ Theory of Interpersonal Behaviour (TIB).

The refined model of the TIB proposed by Jackson [22] indicates that interpersonal human behaviour may be primarily influenced by four types of factors: attitude, social factors, affect, and habits. According to the TIB, emotions come under the umbrella of *affect* and thus influence interpersonal human behaviour. Therefore, according to the TIB, in human-human interactions, both *attitude* and *emotions* are factors influencing engaging behaviour.

We show that even in a human-robot setting, attitude and emotion are indeed highly correlated with engaging behaviour. Their correlation patterns provide various insights as discussed below and in future sections, and justify the use of attitude and emotions to make inferences about engaging behaviour.

To obtain inferences about attitude and emotion from the multimodal data and Big Five trait scores, we use the Interpersonal Circumplex (IPC) [23, 24] as an interface between personality and the two influencers of behaviour, attitude and emotion. Previous work [21] with the IPC gives a procedure to project the Big Five personality traits onto the IPC. Russell [25] proposes a circumplex of emotion corresponding to the IPC. Methods like the Interpersonal Check List (ICL) [26] and Interpersonal Adjective Scales (IAS) [27] exist, that build upon IPC to measure the perception of attitudes [28].

We contribute a novel mechanism to go from Big Five personality traits to attitude and emotion. The mechanism exploits the interpretability of the IPC in behaviour, attitude, and emotion evaluations. The Big Five traits predicted from multimodal features are grouped as a 5-tuple personality embedding, that is then projected onto the IPC in the sectors corresponding to each trait [21], resulting in five vectors. Measures of attitude and emotion are obtained using the magnitudes and directions of these five vectors. Finally, we obtain behavioural inferences based on the attitude and emotion measures. This completes our pipeline to model engaging behaviour based on the TIB and the IPC.

Prior to this, not much work appears to have been done to utilize personality and behavioural theories in a human-robot interaction setting. Our work gives empirical validation of various psychological results observed in engagement modelling in the following manner.

1. The highest correlation of emotion and attitude with engaging behaviour is observed for extraverted humans, in accordance with observations made by Glas and Pelachaud [1]. Hence, the correlation values we generate can be directly used in any engagement calculation, if the user’s *extraversion* is known.
2. The correlation of emotion and attitude scores to engaging behaviour is positive when these are generated using personality labels that the users give to themselves. This corroborates the TIB’s claim that self-concept also determines behaviour [14]. It also suggests that if we know a human’s perception of their personality, we can determine their behaviour with greater accuracy.
3. Wegrzyn et al. [29] establish a correspondence between emotions and various parts of the human face. The TIB emphasizes the importance of emotions while predicting behaviour. Our pipeline unites these two results by first using facial features extracted from multimodal data to generate emotion scores, and then using the same to obtain engaging behaviour.

The TIB [14] was predominantly intended to model human-human interactions. It has been studied, evolved, and proven over the years. Its empirical validation in the HRI setting opens up the possibility of improved psychological theories of human-agent interactions.

We build the pipeline to model engagement incrementally, by making a set of five observations (labelled O1 through O5) and validating them through a Support Vector Machine (SVM) and other statistical measures (e.g., Pearson Correlation, F1 score, etc.). Each observation logically leads to the next (Refer to Section 4). The observation O1 and the corresponding tests relate to predicting engaging behaviour using physiological and first-person vision features extracted from the MHHRI dataset (Table 3 in Section 4.1). The tests corresponding to observation O2 relate to predicting the Big Five traits using the same features (Table 4 in Section 4.2). The tests conducted for these two observations build upon the work by Celiktutan et al. [18]. Observation O3 analyses the relationships between Big Five personality traits and engaging behaviour (Table 5 in Section 4.3).

The novel contributions (described below with their corresponding observations) of this work are the next steps of the pipeline.

1. O4(i): A method to obtain attitude and emotion scores from the Big Five personality projections on the IPC (Section 3.4 and Fig 5a).
2. O4(ii): Quantifying the correlation between attitude and emotion scores with engaging behaviour (Tables 5, 6 in Section 4.4).
3. O5: An end-to-end pipeline to obtain behavioural inferences from multimodal data, in HRI settings (Fig 5b in Section 4.5).

The pipeline is useful in settings where the robot has an input of multimodal input of the interacting human, and is required to judge the level of engagement. The pipeline leverages various psychology models that already exist for both behaviour(TIB) and personality(IPC) in human-human interactions and applies them to a human-robot setting. The empirical validation of the TIB in HRI settings opens up the possibility of using our pipeline to model other behaviours such as decisiveness, non-compliance etc. The prediction of personality and the mapping of the Big 5 embeddings onto the IPC give further insight into the human-robot interaction [24]. Robotic applications can use our pipeline to gauge the engagement of humans and adapt themselves to accommodate individuals with different attitudes and emotions. Thus, our pipeline is more comprehensive and stands out from other algorithms that use ML classifiers of varying complexity to learn correlations between multimodal input and the engagement of humans [6–8]. The robot implementing our pipeline is able to infer the engagement of the human in the HRI setting, perceive the human’s personality and make detailed inferences about their behaviours.

The rest of the paper is organised as follows: Sections 2.1–2.4, give the required background for the Big Five traits, IPC, TIB and engagement prediction; Section 2.5 describes the MHHRI dataset used. Section 3 gives a pictorial representation and description of the architecture of the pipeline. Section 4 presents the five observations and the results obtained using our pipeline. Section 5 discusses the observations and insights obtained from the results, and Section 6 presents the conclusions. Finally, Section 7 describes the future directions of this work.

## 2 Background

Personality and behaviour are both pivotal concepts in psychology. Simply put, personality is “what we are” while behaviour is “what we do”. Thus, personality is more permanent while behaviour can change based on several factors like values, beliefs, situations, etc. Our pipeline for engagement prediction is firmly based on models of personality and behaviour used for analysing human-human interactions, but applied to human-robot interactions. This section sets the context and the necessary nomenclature to understand the detailed approach described in the next section.

### 2.1 Behaviour

Various theories exist that try to model human behaviour in human-human interactions and try to find the “building blocks” or the factors that lead to certain behaviours. For example, the Theory of Reasoned Action [13] models behaviour based on *attitudes*, *norms* and *intentions*; the Theory of Planned Behaviour [12] builds on the former by adding *control beliefs* and *perceived power* as additional determinants of behaviour.

Much work has been done to employ these theories in the human-robot interaction setting. If a robot can learn the model for a certain behaviour, it may be able to predict the behaviour of a human. For instance, Chai et al. [30] model a student’s willingness to study a subject based on the Theory of Reasoned Action. Piçarra et al. [31] and Sirithunge et al. [32] use the theoretical framework provided by the Theory of Planned Behaviour to design context-aware social robots. These theories are also applied in a variety of other fields like organisational behaviour management [33], online education [34], etc.

**Fig 1:**
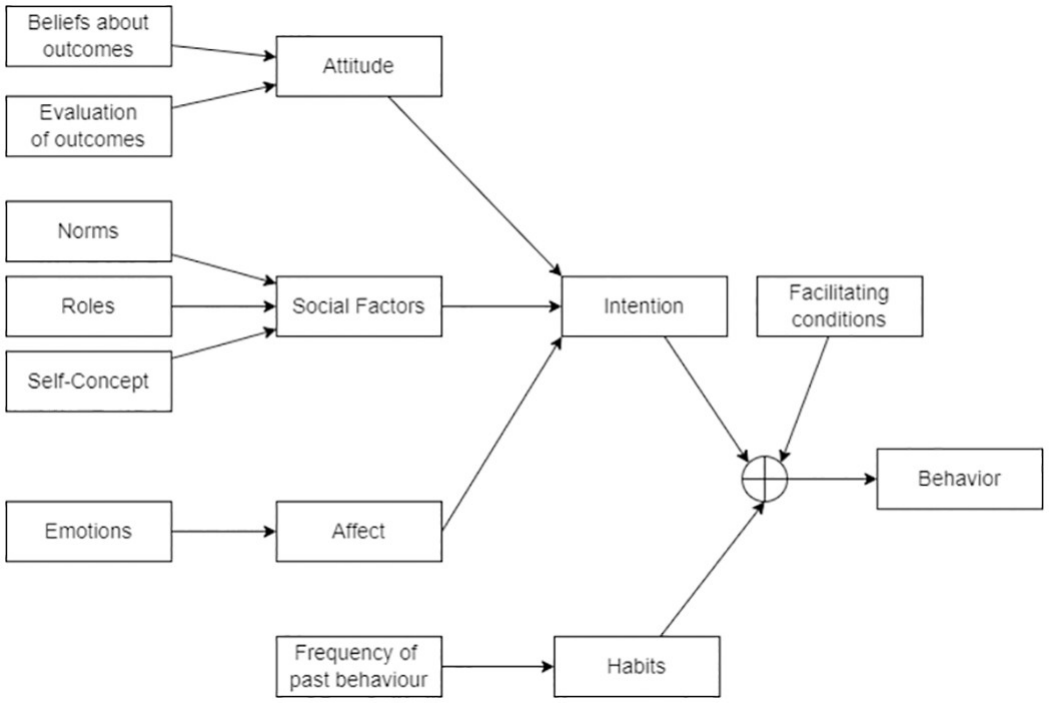
Triandis’ Theory of Interpersonal Behaviour. (Reference: Jackson [22]).

The Triandis Theory of Interpersonal Behaviour (TIB) was proposed by social psychologist Harry Triandis [14] as an integrated model of interpersonal behaviour. According to the TIB (Fig 1), the four types of factors that may primarily influence behaviour include attitude, social factors, affect, and habits. Further, the TIB recognises that emotions play a key role in forming intentions to behave in a certain way [22].

We specifically look at the two factors, *attitude* and *emotion*, of the TIB and use them to build a model for predicting engagement (engaging behaviour) in HRI settings. *Attitude* refers to the individual’s beliefs about the outcome of certain behaviour. *Emotions* refer to the emotional responses to a situation that determines the human’s behaviour.

### 2.2 Personality

Personality is expressed as *traits*, which are relatively enduring characteristics. The most accepted way of measuring traits is through personality tests wherein people self-report their characteristics. Two popular personality tests include the “Myers-Briggs Type Indicator (MBTI)” [35] and the “Five-Factor (Big Five) Model of Personality” [36]. Studies have shown that the MBTI is not reliable or valid as it does not relate to other measures of personality or behaviour [37]. On the other hand, a large body of research [38–40] has supported the Big Five model.

The Big Five model is based on five fundamental underlying trait dimensions namely: *openness* to experience, *conscientiousness*, *extraversion*, *agreeableness*, and *neuroticism*. These five dimensions are “Stable across time, cross-culturally shared, and explain a substantial proportion of behaviour” [41]. The Big Five model is apt for this work due to its close relation to behaviour. For instance, humans with high *openness* and *conscientiousness* have been known to win more points in video games; those with low *agreeableness* tend to reach further levels [42]. Many studies on psychological disorders and mental health [43] utilise behavioural analysis based on the Big Five dimensions.

There is a varied set of techniques in (Big Five traits) personality prediction. Some of them include the prediction of Big Five traits from brain signals [44], handwriting [45], digital footprints on social media [46–48], etc.

### 2.3 Interpersonal Circumplex (IPC)

In the HRI setting, much work has been done on modelling behaviour independent of personality. However, the modelling of behaviour using personality cues, backed by a theoretical framework such as the TIB, seems to be sparsely explored. Salam et al. [11] describe a method to use multimodal cues integrated with Big Five traits for predicting engaging behaviour. Automatic regressors are trained and used to predict participants’ Big Five personality traits. These predictions are combined with individual and interpersonal multimodal features to train the engagement classifier.

We enrich the above process by modelling engaging behaviour based on the TIB. To bridge the dimensions of personality and the factors affecting behaviour (as per the TIB), we use the Interpersonal Circumplex (IPC) [23].

Instead of using a scale with two opposing poles, the IPC provides a circular classification system or *circumplex* on the interface of traits and behaviour. The top and bottom of the IPC are labelled *dominant* and *submissive* respectively, which are mutual opposites. Thus the vertical axis corresponds to *assertiveness* or control. The labels on the left and right are *hostile* and *friendly* respectively. Thus the horizontal axis corresponds to *affection* or warmth.

**Fig 2:**
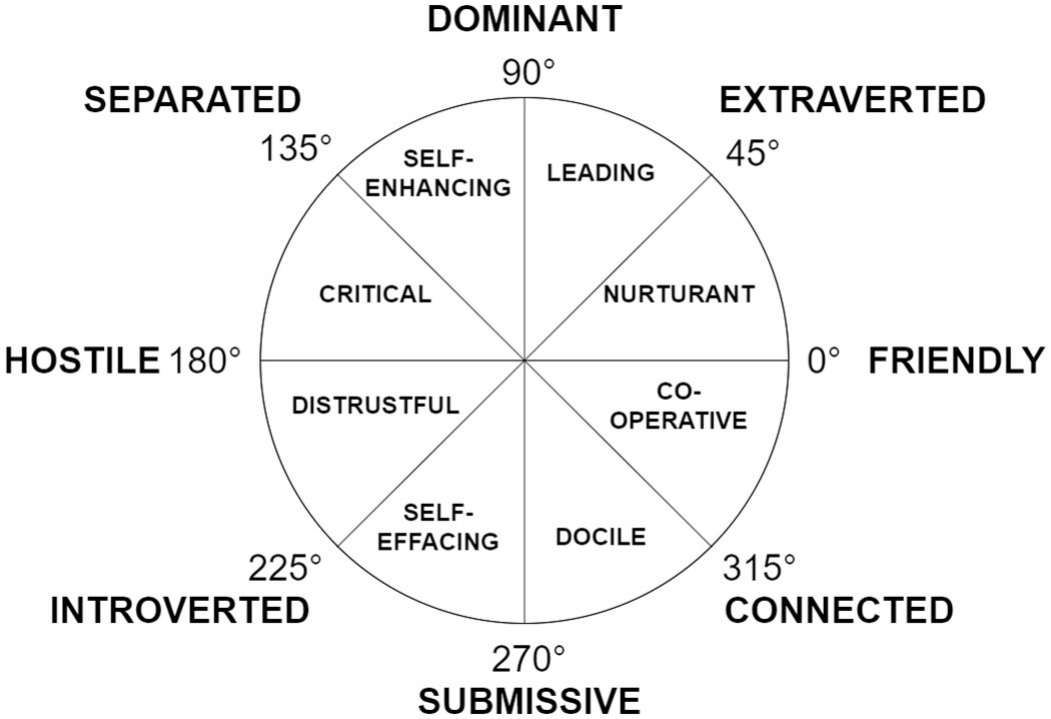
Interpersonal Circumplex. The field nature is visible in the positioning of similar behaviours next to each other. (Extracted from Orford [49]).

The most utilitarian feature of the IPC is that it follows a field-like nature: each behaviour is similar to the one adjacent to it and reciprocal opposite to it [49, 50], as shown in Fig 2. Though the IPC has been around for a while, interest in it has increased recently due to its applicability in computational psychology. Du et al. [21] give a mechanism to project the dimensions of the Big Five traits onto the IPC. We use this mechanism, with insights from Ansell and Pincus [51] and Orford [49] to develop a method to relate emotion and attitude to the Big Five traits projected onto the IPC.

### 2.4 Engagement

Engagement in human-human interactions is defined by Sidner and Dzikovska [52] as: “The process by which two (or more) participants establish, maintain and end their perceived connection.” In the case of human-robot interactions, some widely accepted definitions of engagement are given by O’Brien and Toms [53], based on user experience and by Bickmore, Schulman and Yin [54], based on the involvement a user chooses to have with a system.

Different definitions are applicable in different HRI settings. Thus many techniques exist under under the umbrella of engagement prediction in human-robot interactions. Hussain et al. [2] define student engagement based on their participation in Virtual Learning Environment(VLE) activities. They classify low-engagement students based on features like the number of clicks, differential investment of time, etc. Bárbaro et al. [55] define engagement based on the time users spend on a mobile app and predict when a user may get disengaged via different numerical models on features like the number of interactions, type of interactions, etc.

Another notion about engagement in the HRI setting is that—it falls within three categories: behavioural, cognitive, and affective/emotional (Fredericks, Blumenfeld, and Paris [56]). The definitions for engagement discussed above, fall into one or more of these interrelated categories. However, as observed by Ladd and Dinella [57]:

“Based on current research and understanding, we don’t know how the three types of engagement interact, and we are not certain which antecedents are linked to which types.”

Even so, it is generally noted that the affective/ emotional component of engagement is embodied by enjoyment [58]. If the human and the robot in the interaction feel that they “enjoyed the interaction”, it is a result of the interplay of behavioural and emotional engagement. This is the survey question used to assign labels for engaging behaviour in the MHHRI dataset (engagement index), as mentioned in Section 2.5.

Thus, our work looks at predicting engagement as a “behaviour” displayed in the HRI setting. The pipeline incorporates attitude and emotions, capturing the flavours of different engagement categories. We verify our pipeline for behaviour prediction in human-robot interactions by checking the model’s accuracy for engaging behaviour. The table below describes some of the previous important works related to engagement prediction, including the definition of engagement used, dataset, strengths and weaknesses.

Like the approaches summarised in Table 1, our pipeline for engagement prediction is also data-driven. However, unlike the majority of the algorithms that directly learn correlations from verbal/multimodal cues to engagement, our pipeline feeds the data through many behaviour and personality models like TIB and IPC and finally gives richer engagement inferences. The initial steps of our pipeline take inspiration from Celiktutan et al. [18] as we also predict and utilise the personality traits of the interacting human. Additionally, our pipeline analyses engagement as a behaviour, through the lens of TIB and gives more accurate engagement prediction scores than Celiktutan et al. [18].

**Table 1:** Summary of data-driven models for engagement prediction, their strengths and weaknesses.

Article	Engagement Definition	Strength	Weakness
Bohus and Horvitz [6]	Whether a user intends to engage in an interaction with a system	Multimodal data-driven, uses ML algorithms not just statistics	Does not use body pose, eye gaze, context and longer-term memory features
Castellano et al. [7]	Positive feeling of the users after every interaction with affect sensitive novel platform iCat	Context-sensitive, non-verbal cues and information about the user’s task also used as features	Does not consider personality traits and behaviours of users
Foster et al. [8]	Whether that user currently requires attention from the system	Data-driven, higher accuracy and robust feature engineering	Binary classification completely based on sensor information; Doesn’t account for emotions and attitudes of users
Celiktutan et al. [18]	If user enjoyed the interaction with robot	Takes user personality into account, uses data from 6 modalities	Not based on any behavioural/personality models; Directly predicts engagement from data

### 2.5 MHHRI dataset

The dataset used is the Multimodal Human-Human-Robot-Interactions (MHHRI) dataset [18] which was created to study personality and its relationship with engagement in human-human interactions (HHI) and human-robot interactions (HRI). The dataset has two parts corresponding to HHI and HRI. The HHI part comprises multimodal data collected during dyadic interactions between two human participants. The HRI part comprises multimodal data collected during triadic natural interactions between the same two humans and a small humanoid robot.

Data were acquired from a total of 18 participants and recorded throughout 12 independent interaction sessions. Each session was divided into numerous video clips, where every clip was recorded using a set of sensors including two first-person vision (FPV) cameras (also known as egocentric cameras), two Kinect depth sensors and two physiological sensors. This resulted in approximately 4 hours and 15 minutes of fully synchronised multimodal recordings.

The annotations provided in the dataset include:

1. Self-assessed Big Five personality traits (self labels) obtained by having participants fill in a questionnaire, for assessing their personality traits.
2. Engagement indices are obtained by having participants fill in a questionnaire about their perceived engagement.
3. Acquaintance assessments for the Big Five personality traits (acquaintance labels) obtained by having participants fill in a questionnaire, to assess the personality traits of the other participants partaking in the study.

The reasons the MHHRI dataset is well suited for this work are:

1. It incorporates human-human interaction and human-robot interaction, unlike previous multimodal datasets that exclusively focus on either human-human interaction or human-robot interaction.
2. In addition to the static, third-person vision cameras, the conversations are recorded using dynamic, first-person vision cameras.
3. It offers both personality and engagement labels.
4. It provides fully synchronised recordings of six different data modalities ranging from visual to physiological.

The data collected from first-person vision cameras and physiological sensors were primarily used in our work.

## 3 System architecture

**Fig 3:**
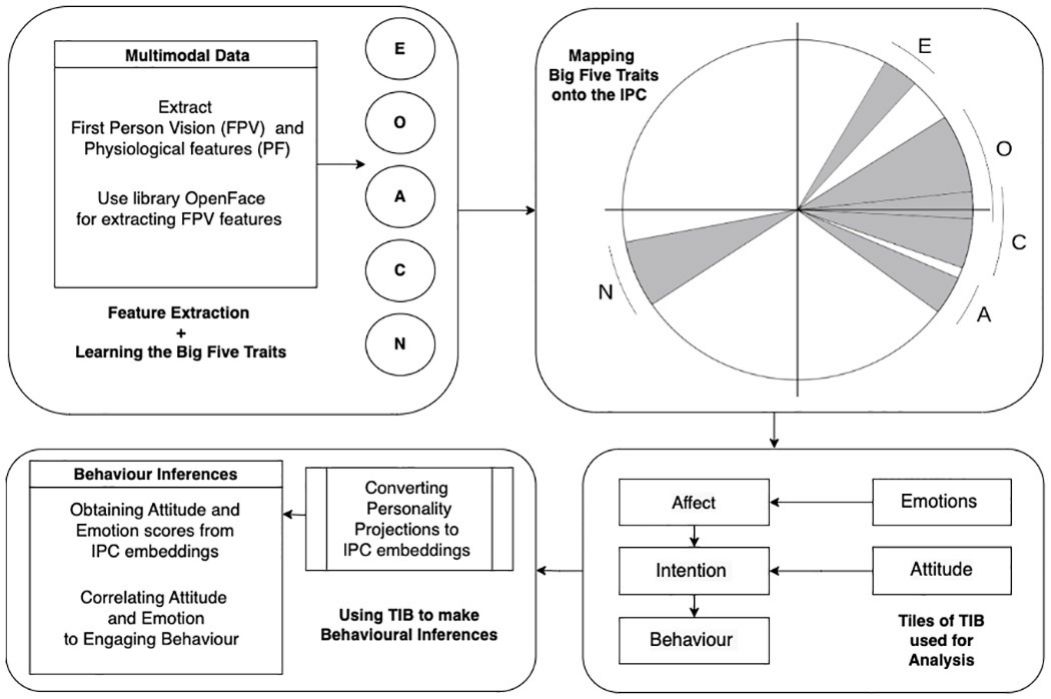
Representation of the proposed pipeline: From multimodal data to behaviour inferences. E: Extraversion, O: Openness. A: Agreeableness, C: Conscientiousness, N: Neuroticism.

Fig 3 shows a high-level view of the designed end-to-end pipeline which consists of modules dedicated to:

1. **Feature Extraction + Learning the Big Five Traits**: This module performs the feature extraction from multimodal data and the features extracted are utilised to train SVM for learning the Big Five Traits.
2. **Mapping the Big Five Traits onto the IPC**: This module creates a projection map of the Big Five Traits onto the IPC.
3. **Tiles of TIB used for analysis**.
4. **Using TIB to make Behavioural inferences**: This module is responsible for inferences of behaviour, attitude and emotion using the TIB.

### 3.1 Feature extraction from multimodal data

The MHHRI dataset consists of multimodal data of dyadic interactions between human-human and triadic interactions between human-human-robot. To learn from the multimodal data, we need to extract the relevant features (or cues). We primarily deal with two modes of data while extracting features—physiological and first-person vision (FPV). The physiological features chosen for each person and their extraction processes are similar to Celiktutan et al. [18]. However, the FPV features obtained from the first-person vision cameras and their extraction processes used in this work are quite different compared to Celiktutan et al. [18].

1. *Physiological Features*:
  There are five physiological measurements—electrodermal activity (EDA), skin temperature, and the acceleration of the wrist along the three spatial axes. For each physiological measurement, we consider the (i) maximum, (ii) minimum, (iii) mean and (iv) standard deviation. In addition to this, we also consider the mean absolute difference of (v) first-order and (vi) second-order derivatives of the measured values in a single clip. Thus, each physiological measurement adds six features to the feature space. Therefore, we have a total of 5 physiological measurements × 6 features = 30 physiological features per clip.
2. *First Person Vision Features*:
  The clips are from the camera worn by the first person in the interaction. Thus, the first-person vision features obtained from each clip correspond to the second (other) person, sitting across, during the interaction. We use the FaceLandmarkImg and FeatureExtraction functionalities of the OpenFace tool [59] to obtain the first-person vision measurements from each clip. OpenFace generates over 150 measurements for each timestamp in a given clip. From them, we select measurements related to the location of the head, eye gaze directions, head pose, and facial action units (AUs) that describe human facial expressions (eyebrows, lips, etc.), resulting in a total of 31 measurements per clip. This selection is based on insights from Celiktutan et al. [60] and Wegrzyn et al. [29]. Six features are added to the feature space for every measurement, as in the case of physiological features. Therefore, we have a total of 31 first-person measurements × 6 features = 186 first-person vision features per clip.

### 3.2 Learning Big Five traits from multimodal data

The problem of predicting the Big Five traits and the engagement index from multimodal data may be considered a classification task.

We binarize the Big Five labels using the mean values of the distribution as a threshold. As the MHHRI dataset only has data from 18 subjects, we use a traditional and popular machine learning approach—Support Vector Machine (SVM) instead of any deep learning architectures. This SVM uses a radial basis function to perform the predictions. Big Five trait predictions involve training and testing a separate classifier for each of the traits and engagement indices.

The metric used for evaluation is cross-validation, more specifically—leave-one-subject-out cross-validation. We train the classifiers on the data obtained from 17 users and consider one user as a test subject. This is done iteratively over the entire dataset, to ensure better generalizability of the classifiers.

### 3.3 Mapping Big Five traits to the IPC

In this step, we convert the personality embedding into an IPC embedding. The problem boils down to converting Big Five scores to a set of five vectors in a 2-dimensional circular plane. Previous work by Du et al. [21], and Ansell and Pincus [51] give the sector angles (lower bound, upper bound and the mean angle) for every trait of the Big Five over the IPC. We take the mean angles (given in Table 2) as the directions of the five vectors corresponding to the Big Five traits. We take the magnitude of each vector as proportional to the score of the corresponding Big Five trait. These five vectors, when aggregated, form the IPC embedding. $v_{p}=\left[v_{p_{e}},v_{p_{a}},v_{p_{c}},v_{p_{n}},v_{p_{o}}\right]$, is the 5-tuple *personality embedding* consisting of the vectors $v_{p_{e}},v_{p_{a}},v_{p_{c}},v_{p_{n}},v_{p_{o}}$, whose magnitudes are the scores (out of 10) for the Big Five traits *extraversion*, *agreeableness*, *conscientiousness*, *neuroticism* and *openness* respectively.

**Table 2:** The upper, lower, and mean angles used for projecting the Big Five traits over the IPC. (Extracted from Du et al. [21]).

Big Five traits	Extraversion	Agreeableness	Conscientiousness	Neuroticism	Openness
Lower Bound Angle (in degrees)	48.1	323.2	340.1	191.0	357.0
Upper Bound Angle (in degrees)	60.0	336.3	6.2	213.8	32.9
Mean Angle (in degrees)	54.2	329.8	353.1	202.6	15.7

To obtain the IPC embedding from the 5-tuple personality embedding, we first resolve each of the five vectors into sine and cosine components. The final horizontal component is created by aggregating the cosine projections of the Big Five vectors in the 5-tuple personality embedding:

$$\begin{array}{c} v_{ipc_{h}}=\sum_{\forall t\in \{e,o,a,c,n\}}^{}v_{p_{t}}\;\text{cos}\;m_{t} \end{array}$$ (1)

The final vertical component is created by aggregating the sine projections of the same:

$$\begin{array}{c} v_{ipc_{v}}=\sum_{\forall t\in \{e,o,a,c,n\}}^{}v_{p_{t}}\;\text{sin}\;m_{t} \end{array}$$ (2)

where *m*_*e*_, *m*_*o*_, *m*_*a*_, *m*_*c*_ and *m*_*n*_ are the mean angles (in degrees) associated with the Big Five traits on the IPC (refer Table 2). *v*_*ipc*_ is the 2-dimensional vector or the IPC embedding of the personality with the attributes, $v_{ipc_{v}}$ and $v_{ipc_{h}}$ which denote the final horizontal and vertical components respectively. We normalize the vertical and horizontal components such that their squared sum adds up to 1 (L2 normalization). The final IPC embedding *v*_*ipc*_ is initialised as the ordered pair $\langle v_{ipc_{h}}^{\prime },v_{ipc_{v}}^{\prime }\rangle$ where $v_{ipc_{h}}^{\prime }$ and $v_{ipc_{v}}^{\prime }$ are the L2 normalized components.

### 3.4 Relating the IPC embedding to Triandis’ Theory of Interpersonal Behaviour and engagement

**Fig 4:**
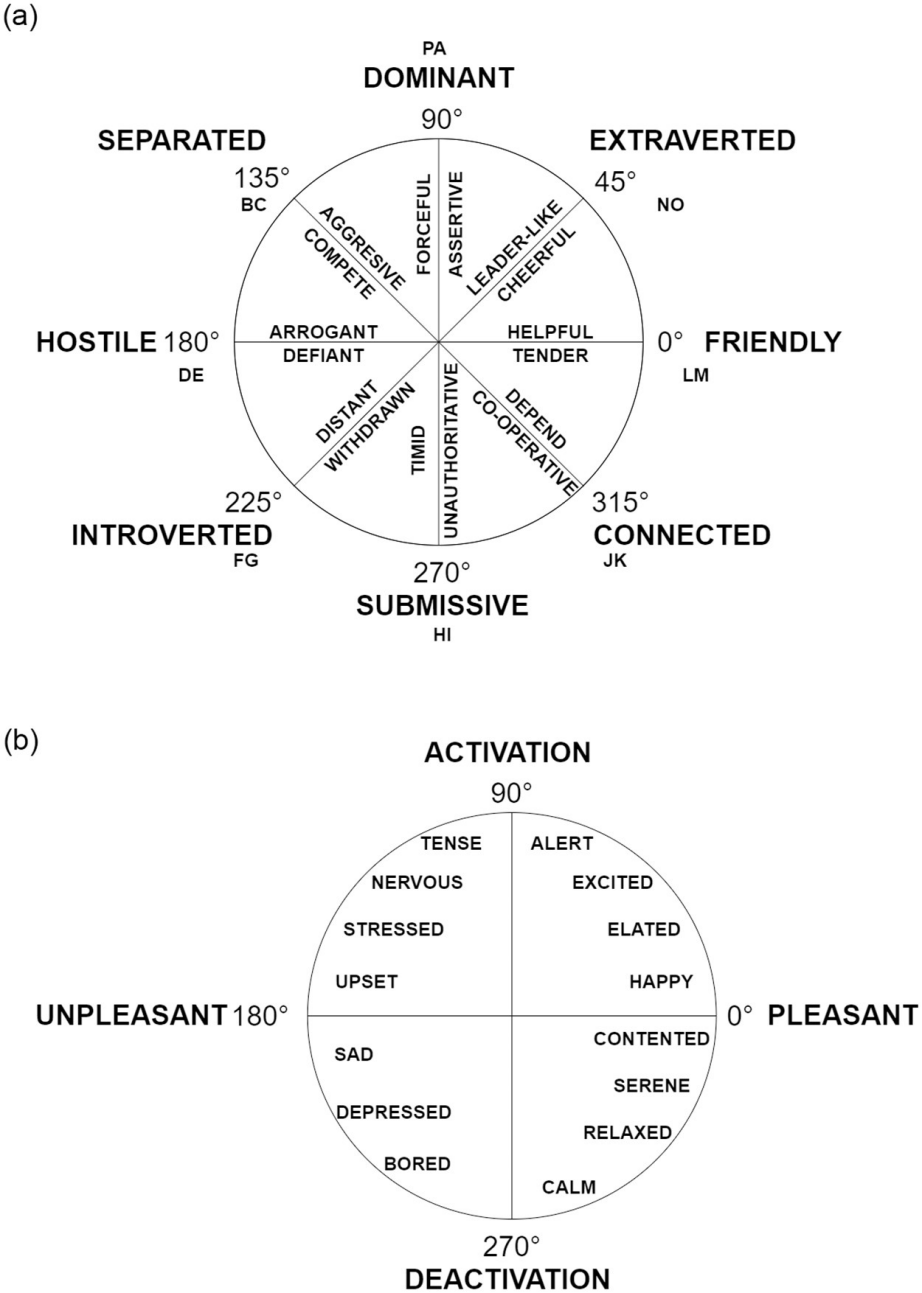
Relating emotion and attitude to behaviour using IPC. (**a**) IPC for attitude. (Extracted from Dermouche and Pelachaud [28].) (**b**) IPC for emotion. (Extracted from Russell [25].).

We now utilize the IPC to relate components of the TIB to engaging behaviour. The two factors of the TIB that we concentrate on are attitude and emotion. The IPC in its original form (as shown in Fig 2) is a classification system for behaviours. However, Dermouche and Pelachaud [28] modified the IPC to classify attitudes as shown in Fig 4a. Similarly, Russell [25] proposed a system that models human emotions according to the IPC. The emotions are arranged on the IPC as shown in Fig 4b.

As the IPC is interpretable both in terms of emotions and attitude, any vector on the IPC carries information about that particular human’s attitude as well as emotion. Thus the IPC embedding we obtain as previously, $v_{ipc}=\langle v_{ipc_{h}},v_{ipc_{v}}\rangle$, is representative of the human’s attitude as well as emotion. We take the final horizontal component $v_{ipc_{h}}$ along the *warm-cold* dimension of the IPC (the *x* axis) to represent emotion. Similarly, we attach the notion of attitude to the *dominant-submissive* dimension (the *y* axis) and hence use the final vertical component $v_{ipc_{v}}$.

This way we can obtain scores for the attitude and emotion components of the TIB. Finally, we investigate the correlations that exist between these scores and the engagement index, showing that the attitude and the emotion of a human indeed determine engagement in the HRI setting.

## 4 Analyses and results

We use the multimodal cues obtained in human-human-robot interactions to model the user’s engaging behaviour. We make five observations (O1 through O5) and scrutinize them sequentially to build a pipeline that models engaging behaviour. Thus, the corresponding analyses verify different regions of the pipeline (refer Fig 3).

Many concepts used in the pipeline, like Big Five traits, the IPC, and the TIB, were originally proposed for human-human interactions. The analyses that follow are aimed to verify their applicability in human-robot interactions as well. Thus, the observations are validated for both the human-human interactions (HHI) and human-robot interactions (HRI) and the values are shown to be comparable.

The following evaluation metrics are used for the analyses:

1. F1 Score: This is a performance metric calculated by considering Recall and Precision into account. (See [61] for a discussion on various metrics to calculate performance).
2. Pearson Correlation: This indicates how the two parameters are correlated. The value of this metric ranges from -1 (total negative correlation) to 1 (total positive correlation). (See [62] for a discussion on Pearson Correlation).

The analyses were performed on Google Colab (with a Python 3 Google Compute Engine backend).

### 4.1 Predicting engaging behaviour directly from multimodal data

**O1**: Engagement Index can be accurately predicted directly from multimodal data features.

Previously, attempts have been made to use multimodal features directly for the task of engagement classification [6–8]. Thus, our first step is to directly predict engagement from features extracted from the MHHRI dataset. The physiological features and first-person vision features extracted (refer Section 3.1) are scaled with the help of StandardScaler, a Scikit-learn [63] library function for scaling the features. Engagement labels are binarised with respect to mean value, and prediction is carried out as per the process given in Section 3.2.

We verify that the physiological and first-person vision features yield good cross-validated F1 scores for engagement classification (see Berrar [64] for a discussion on cross-validation methods). Table 3 gives the F1 scores after cross-validation which are better than those of Celiktutan et al. [18], for all four scenarios. However, this analysis does not consider the personality of the user, which is an important factor in behavioural studies [11]. Thus we introduce the Big Five traits into the pipeline.

**Table 3:** Predicting the engagement index from the 30 physiological and 185 first-person vision features of 18 users in HHI and HRI settings. An SVM classifier is used in each case. The values are the F1 scores obtained after performing 18-fold cross-validation. The F1 scores of Celiktutan et al. [18] are presented in brackets for comparison, with improvements shown in bold.

Features	Cross-Validation Scores	
	HHI	HRI
Physiological	**0.71** (0.54)	**0.78** (0.58)
First Person Vision	**0.80** (0.31)	**0.63** (0.59)

### 4.2 Predicting Big Five traits from multimodal data

**O2**: Multimodal features can be used to predict personality in human-human and human-robot interactions.

The physiological and first-person vision features used for this analysis are again extracted as per Section 3.1 and are standardised. The Big Five traits are binarised with respect to the mean value for the corresponding trait.

Acquaintance labels are the Big Five trait scores assigned to users by those who interacted with them. Self labels are the scores users assign to themselves. We predict the Big Five traits from the extracted multimodal features under four scenarios: in the human-human setting and human-robot setting, first using self labels and then using acquaintance labels for both settings.

In Table 4, we witness better results (as marked in bold) as compared to those presented in Tables 8 and 9 of Celiktutan et al. [18] in both self and acquaintance labels for most traits in the Big Five traits. We then proceed to utilize the Big Five predictions in modelling engaging behaviour.

**Table 4:** F1 scores for the Big Five traits predicted from the multimodal features in both HHI and HRI settings, with self and acquaintance labels. The F1 scores by Celiktutan et al. [18] for the high class are presented in brackets for comparison. The scores which are an improvement are shown in bold.

Big Five traits		PF		FPV	
		HHI	HRI	HHI	HRI
Extraversion	Self	**0.80** (0.68)	**0.76** (0.53)	**0.81** (0.64)	**0.68** (0.59)
	Acquaintance	**0.76** (0.04)	**0.73** (0.27)	**0.66** (0.42)	**0.58** (0.53)
Agreeableness	Self	**0.74** (0.59)	**0.70** (0.38)	**0.76** (0.36)	**0.57** (0.39)
	Acquaintance	**0.71** (0.00)	**0.75** (0.42)	**0.74** (0.54)	**0.61** (0.37)
Conscientiousness	Self	**0.81** (0.37)	**0.80** (0.37)	**0.80** (0.31)	**0.59** (0.37)
	Acquaintance	**0.72** (0.34)	**0.71** (0.21)	**0.84** (0.31)	**0.60** (0.42)
Neuroticism	Self	**0.74** (0.27)	**0.87** (0.41)	**0.84** (0.28)	**0.78** (0.30)
	Acquaintance	**0.65** (0.51)	**0.65** (0.62)	**0.83** (0.35)	**0.58** (0.42)
Openness	Self	**0.79** (0.46)	**0.80** (0.63)	**0.82** (0.62)	0.55 (0.64)
	Acquaintance	**0.76** (0.41)	**0.76** (0.56)	**0.72** (0.65)	**0.58** (0.52)

### 4.3 Recognizing patterns in Big Five traits and engagement index

**O3**: Engagement index is not strongly correlated to the Big Five traits predicted, in human-human interaction (HHI) and human-robot interaction (HRI) settings.

Hashimoto and Oshio [65] suggested the correlation of the Big Five traits, especially *agreeableness* and *extraversion*, to the components of IPC. Since we have the ground truth values for the engagement index as well as the Big Five traits, we quantify this correlation. If the correlation is high, then engaging behaviour can be inferred from the predicted Big Five traits, in any interaction.

In Table 5, in the case of self labels, *extraversion* and *agreeableness* are indeed slightly correlated to engaging behaviour along with *neuroticism*. However, in the case of acquaintance labels, the correlation is very low to negative. Thus, a direct correlation is insufficient to infer engaging behaviour from the user’s Big Five traits scores.

**Table 5:** The Pearson Correlation coefficient quantifies the correlation between ground truth engagement indices and the predicted Big Five trait scores in the HRI setting.

Big Five trait	Self labels	Acquaintance labels
Extraversion	0.08	0.06
Agreeableness	**0.29**	-0.13
Conscientiousness	0.05	0.12
Neuroticism	**0.48**	-0.25
Openness	0.03	-0.42

In our next analysis, we bring about meaningful correlations between Big Five traits and behaviour (specifically engaging behaviour) based on the Triandis Theory of Interpersonal Behaviour.

### 4.4 Modelling the Triandis Theory of Interpersonal Behaviour for engagement

**O4**: The Big Five traits can be mapped onto the IPC, and: (i) relations can be established between emotion, attitude and IPC using component analysis; and (ii) segregation of the user group into two classes, *extroverts* and *introverts*, results in observable correlations of engagement with emotion and attitude.

From the Big Five trait scores we obtain attitude and emotion scores, as given in Section 3.4. Emotion is associated with the warm / *agreeableness* axis (the *x* axis) of the IPC, and attitude is associated with the assertiveness axis (the *y* axis).

We proceed to check if engagement shows a better correlation with the attitude and emotion scores obtained from the Big Five trait scores. However, the results presented in Table 6 are still not satisfactory. A positive correlation exists only between the engagement index and assertiveness. Thus, attitude and emotion scores cannot be used directly to infer a user’s engagement.

**Table 6:** Correlation matrix of the IPC components with the engagement index using ground truth self and acquaintance labels in the HRI setting.

	Self labels	Acquaintance labels
Warm / Agreeableness (Emotion)	-0.28	-0.04
Assertiveness (Attitude)	-0.29	**0.27**

Segregation of the users into introverts and extroverts is done by taking the mean of the *extraversion* trait as the threshold for division. We compute the correlations for both groups with ground truth self and acquaintance labels. The correlations are presented in Table 7. Highly engaging individuals show a positive correlation to attitude and emotion scores. Here positive correlation implies that warm and assertive individuals are more likely to be engaging as well.

**Table 7:** Correlation values of the IPC components with the engagement index using ground truth self and acquaintance labels for introverts and extroverts in the HRI setting.

	Introverts		Extroverts	
	Self	Acquaintance	Self	Acquaintance
Warm / Agreeableness (Emotion)	**0.39**	-0.33	-0.27	**0.02**
Assertiveness (Attitude)	**0.56**	**0.26**	**0.34**	**0.16**

Table 8 presents further insights into the relationship between engagement, attitude and emotion, for extraverted and introverted humans. Thus, after segregation, both attitude and emotion scores can be utilized to infer engaging behaviour.

**Table 8:** Observations from correlation patterns of engagement with attitude and emotion, after segregation into extroverts and introverts. A highly engaging individual would show a positive correlation with attitude and emotion scores.

	Introvert	Extrovert
Self	Strong positive correlations for both attitude and emotion components. Validates the presence of Self-Concept tile in TIB.	Positive correlation for attitude. Partial validation.
Acquaintance	Positive correlation for attitude. Partial validation.	Weak positive correlations for attitude and emotion.

The TIB suggests that attitude and emotion may influence interpersonal behaviour in the human-human setting [22]. Our results indicate that attitude and emotion indeed influence engaging behaviour even in the human-robot setting.

### 4.5 Making behavioural inferences using the IPC

**O5**: The IPC embeddings of users with moderate to high engagement indices lie in the region corresponding to −45° to 90° in the IPC.

**Fig 5:**
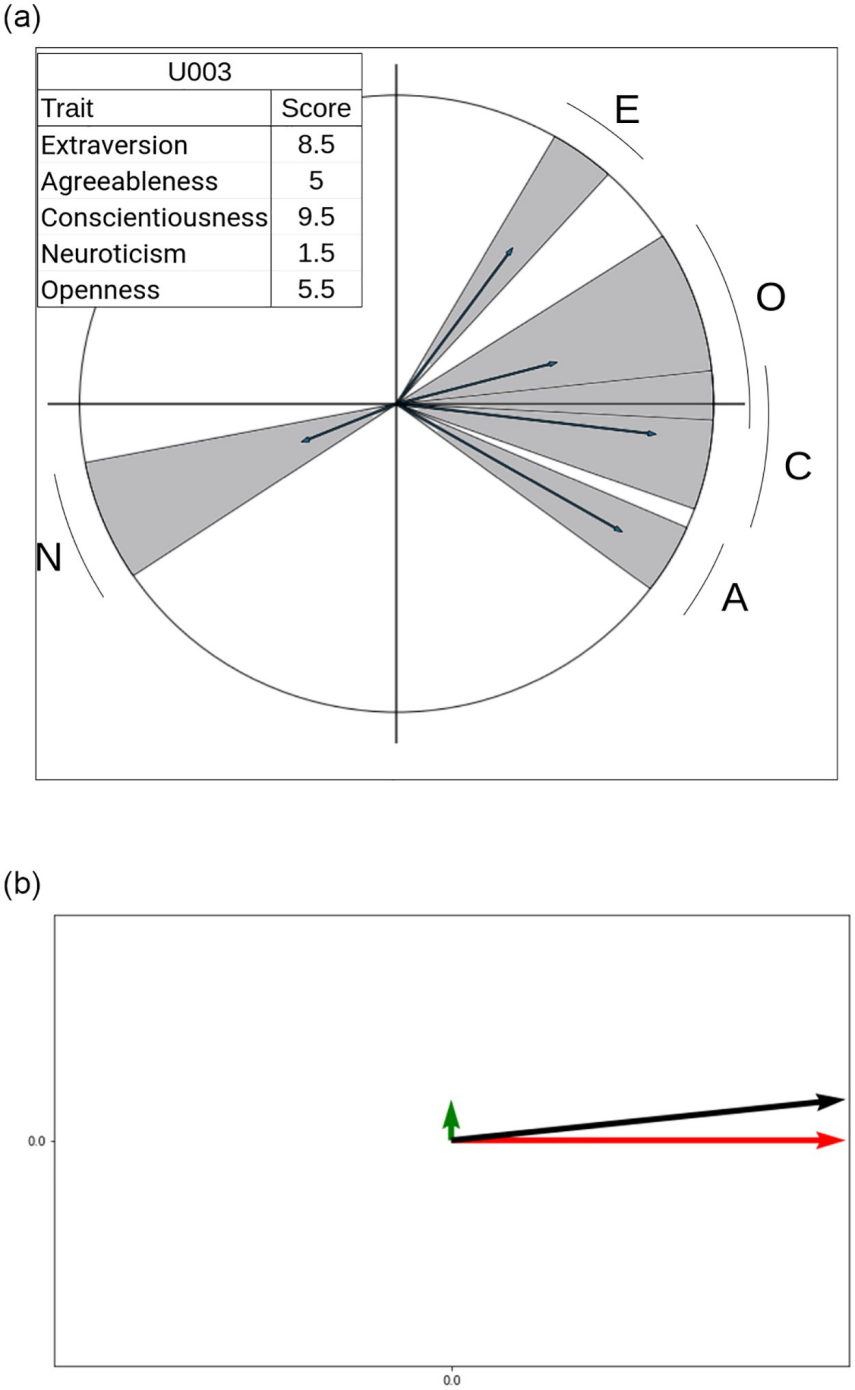
The horizontal component of the IPC embedding gives a measure of emotion and the vertical component, a measure of attitude. (**a**) Projection of the personality embedding (Big Five trait scores) onto the IPC for user U003 in the HRI setting. (**b**) Representation of the relation of engaging behaviour to attitude and emotion for user U003, whose engagement index is 5 (out of 10), in the HRI setting.

From the correlations obtained in Section 4.4, it is expected that users with higher engagement indices have higher positive scores for attitude and emotion. This also implies that they have higher *openness* and *agreeableness* scores before aggregation. Thus, we can say that if a user is perceived as engaging, their IPC embedding is in areas corresponding to these traits (−45° to 90°). Fig 5a shows the projection of the personality embedding onto the IPC for one user (U003 in the MHHRI dataset).

U003 is an extrovert (*extraversion* score of 8.5 out of 10) and has a moderate engagement index (5 out of 10). The IPC embedding for user U003 is shown in Fig 5b. The vector along the *x* axis (shown in red) is the aggregated horizontal component $v_{ipc_{h}}^{\prime }$ which represents an emotion score of 0.98. The vector along the *y* axis (shown in green) is the aggregated vertical component $v_{ipc_{v}}^{\prime }$, which represents an attitude score of 0.18. The resultant vector (shown in black) is the IPC embedding *v*_*ipc*_ for U003, whose direction is along 10.38°. This lies in the expected region from −45° to 90°.

Further behavioural inferences can be made about U003 by looking at the direction and magnitude of their IPC embedding. In this case, U003 is likely to be supportive and show consoling and nurturing behaviour. Similarly, behavioural inferences are obtained for other users.

## 5 Discussion

The first analysis that we performed was the direct prediction of engagement indices from multimodal features. The evaluation metric that we followed was leave-one-subject-out cross-validation, as done by Celiktutan et al. [18]. As Table 3 shows, we obtained better cross-validation scores than Celiktutan et al. [18] in all four scenarios.

There is a considerable difference in accuracy when first-person vision features are used in the HHI and HRI settings. In the HRI setting, the accuracy perhaps falls because the human is involved in a triadic interaction with a robot and another human, dividing his attention. In the HHI setting, the human faces directly towards the camera while interacting with another human, making the first-person vision features more precise.

The next analysis was to predict Big Five traits with the help of multimodal features. Table 4 shows the analysis results, and the values in bold indicate the improvement in cross-validation scores over Celiktutan et al. [18]. In both HHI and HRI settings, *extraversion*, *neuroticism* and *openness* are seen to be classified better with self labels.

Following this, we analyse the correlation patterns between the engagement index and the Big Five traits. Henceforth, we focus our analyses on the HRI setting, as it is our primary area of interest. Table 5 shows the results of the analysis. We observe that *agreeableness* and *neuroticism*, calculated through self labels, show a significant correlation with the engagement index. *Openness*, when calculated from acquaintance labels, also shows a high correlation with the engagement index as shown in Table 5. This observation is in accordance with Kang et al. [66], who suggest that agreeable people show strong self-reported rapport (engagement).

Further, we also analysed the correlation between attitude and emotion factors of the TIB with engaging behaviour. We observe a positive correlation with the engagement index only for attitude scores obtained from acquaintance labels (Table 6). To improve the correlation values, we split the data on attitude and emotion scores into two groups, extrovert and introvert, based on the *extraversion* scores. The split improves the correlation of the engagement index with attitude and emotion, as summarized in Table 8. This verifies, even in the HRI setting, the TIB’s claim that attitude and emotion are factors that may influence behaviour.

Some more insights obtained from the analysis include:

1. The highest correlation of emotion and attitude with engagement behaviour is observed for extraverted humans, in line with observations made by Glas and Pelachaud [1].
2. When emotion and attitude scores are computed using personality labels that the users give to themselves, the correlation to engaging behaviour is higher. This corroborates the TIB’s claim that *self-concept* also determines behaviour [14].
3. The final IPC embedding of highly engaged humans lies in regions corresponding to *openness* and *extraversion* on the IPC. This shows that our pipeline perceives open and extraverted humans as displaying more engaging behaviour, which is as expected [67, 68].

To summarise, the strength of our approach is that it verifies the use of models like Big Five Traits, IPC and TIB, classically used in human-human interactions, as a firm basis to analyse human-robot interactions. However, the pipeline still does not leverage the full advantage of the temporal nature of data present in the MHHRI dataset; no long-term memory features are utilised anywhere. Further, the demographics of the interacting humans, the context of the interaction, and information regarding the nature of the interactions are not utilised in the pipeline. These aspects can potentially serve as areas for future exploration.

## 6 Conclusion

The paper presents a pipeline for predicting human behaviour, personality traits, and tendencies in human-robot interaction using multimodal data. The first-person vision and physiological features are extracted using OpenFace and used to predict the Big Five personality scores. The pipeline utilizes the TIB and IPC to predict engaging behaviour and links personality to behavioural inferences. The positive correlation between human attitude, emotion, and engagement is established, verifying their role as determinants of behaviour in human-robot interaction.

The direct consequence of this work is a ready-to-use pipeline to model the engagement of a human interacting with a robot. For example, it can be used to analyze the engagement of customers interacting with chat-bots and suggest changes in the chat-bot’s interacting style to cater to humans with different attitudes and emotions. In online learning, the pipeline can predict low engagement levels among students and suggest gamification as a way to incentivize them.

Besides, the IPC embedding can be leveraged to make inferences unrelated to engagement, based on the work done by Gurtman [24]. Therefore, this pipeline can help robots better understand the humans they interact with, which is particularly crucial in online learning platforms, chat-bots and assistive robotics [3, 69], etc.

## 7 Future directions

The pipeline can be evolved in the following ways:

1. The pipeline generates features for each clip by aggregating static values over time instances in the clip. The pipeline uses generic classifiers such as SVM and random forest. In the future, LSTMs, which better handle time-series data, may be incorporated to capture dynamics in the clips.
2. A drawback of the MHHRI dataset used in the work is its small sample size of 18 participants, which limits the ability to test hypotheses and analyse results. An avenue for improvement would be to obtain a larger dataset with more participants.
3. The successful validation of the TIB’s attitude and emotion components in human-robot interactions opens up opportunities to use the pipeline for modelling other behaviours, such as inferring caution in users of socially assistive robots [3, 69], measuring non-compliance in class management systems [70], and determining decisiveness in automated interviews [71].
4. The focus of the work was on the attitude [28] and emotion [25] components of the TIB; and connecting the IPC to these factors. If there are advancements in psychology linking the IPC to other components of the TIB such as *habits* or *norms*, their relationship to behaviour in HRI could also be explored.

The proposed pipeline from multimodal data to engaging behaviour is quite easy to generalise and can be used in diverse domains.

## Supporting information

S1 File (ZIP)
